# Virologic and immunologic responses of patients on highly active antiretroviral therapy in a rural community health centre in Limpopo, South Africa: A retrospective study

**DOI:** 10.4102/HIVMED.v20i1.818

**Published:** 2019-05-22

**Authors:** Aniekan Edet, Henry Akinsola, Pascal O. Bessong

**Affiliations:** 1 Department of Public Health, University of Venda, Thohoyandou, South Africa; 2 Department of Family Medicine, Tshilidzini Hospital, Thohoyandou, South Africa; 3 HIV/AIDS and Global Health Research Programnme, Department of Microbiology, School of Mathematical and Natural Sciences, University of Venda, Thohoyandou, South Africa

**Keywords:** HIV, Viral suppression, CD4+ Cell count, Limpopo, South Africa

## Abstract

**Background:**

South Africa has a high HIV burden. Despite increased uptake of persons living with HIV into the South African national antiretroviral therapy programme, the incidence of HIV increased between 2013 and 2016. Studies suggest that increased community viral suppression results in reduced HIV incidence in that community ‘independent of unsafe sexual behaviours and sharing used syringes’.

**Objective:**

The aim of this study was to investigate the viral and immunologic responses of patients, in a rural community health centre in South Africa, to combination antiretroviral therapy (cART) between January 2004 and July 2016.

**Methods:**

This was a retrospective medical record review conducted in Thohoyandou Community Health Centre. Data analysis was done using SPSS 24.0 and Microsoft Excel. The estimates used were 95% confidence intervals, and a *p*-value < 0.05 was considered to be statistically significant.

**Results:**

Analysis was done using 1247 individuals, with 76% of the cohort being female and 98% first-line cART. The proportion of patients with a suppressed viral load (VL) at 6 months post-treatment was 64%, and 72% at 60 months. Fifty-nine per cent had consistent viral suppression over a 6-month period and 14% over at least 54 months. The mean CD4+ cell count at baseline was 227 cells/µL, and 538 cells/µL at 60 months. Multivariate regression analysis revealed that males had poorer immunologic and virologic responses.

**Conclusions:**

Viral suppression in the study population was inferior to the UNAIDS target of 90%. The sustainability of viral suppression, once attained, was also low. These may have a negative impact on HIV transmission.

## Introduction

The prevalence of HIV in South Africa increased from 6.19 to 7.03 million between 2013 and the end of 2015. Despite an increased uptake of persons living with HIV into the national antiretroviral therapy programme over the years, the incidence rate also increased from 340 000 new cases per annum to 380 000 cases per annum during the same period.^1,2,3^ With the scale-up of the antiretroviral therapy programme, the prevalence of viral suppression is expected to increase, with associated reduction in HIV incidence. Studies have suggested that increased community viral suppression results in reduced HIV incidence in that community ‘independent of unsafe sexual behaviours and sharing used syringes’.^4,5^ The Partner study found ‘zero transmission’ when the viral load (VL) at most 1 year before was less than 200 copies/mL.^6^ To curb its high HIV burden, South Africa adopted the UNAIDS ‘90-90-90 targets’. This target aims to attain sustained viral suppression in 90% of all persons receiving antiretroviral therapy by the year 2020.

Over the years, several definitions have been used to define viral suppression and virologic failure, with different definitions recommended for low- to medium-income countries, compared to high-income countries.^7^ The South African National Department of Health (NDoH), however, recommends the use of VL ≤ 50 copies/mL as definition for viral suppression and two VL results > 1000 copies/mL to define viral failure.^8^ Bartlett et al. state that maintaining the VL at ≤ 50 copies/mL is associated with the most durable clinical benefits.^9^ The CD4+ cell response to combination antiretroviral therapy (cART) is, however, more variable. It increases rapidly in the first month of ART by 75 cells/µL–100 cells/µL, with a more gradual response thereafter.^10^ The CD4+ cell count of some patients, however, fails to increase despite viral suppression. Several causes have been suggested for this ‘immuno-virologic discordant’ CD4+ cell response. Insufficient thymic activity and ongoing viral replication, despite good viral suppression, may account for this.^11^ Some studies have investigated the implication of immuno-virologic discordance. Zoufaly et al. found that compared with those whose CD4+ cell count increases with viral suppression, patients with immuno-virologic discordance had increased risk for developing AIDS and other complications, especially in the first 6 months of therapy. Several factors have been shown to be associated with viral suppression, adequate CD4+ cell response and immuno-virologic discordance.^12^

It is known that poor adherence to antiretrovirals is associated with failure to suppress HIV. The best indicator of adherence and response to treatment is virologic response, and more than 90% – 95% adherence is required to achieve viral suppression.^13^ Other factors have also been shown to be associated with a delay in or failure to attain viral suppression. Devey et al.^14^ stated that the age group less than 15 years, male gender, prior ART exposure and being on tuberculosis treatment were associated with an increased risk of virologic failure in South Africa. However, in Swaziland, Jobanputra et al.^15^ found that age group less than 20 years and CD4 count less than 350 cells/µL were associated with virologic failure. In contrast, gender was not an associated factor. Another factor associated with higher incidence of viral failure is a high VL at initiation. A high CD4+ cell count and better clinical stage at initiation are, however, associated with earlier viral suppression.^13^ Finally, Bello et al.^16^ found that a longer duration post-cART initiation, less previous antiretroviral drug use before lifelong cART, higher baseline CD4 cell count and lower baseline VL were associated with 10 years of sustained viral suppression.

## Objectives

The main objective of this study was to investigate the virologic and immunologic responses of patients, in a rural community health centre in South Africa, to cART, as well as the factors that are associated with these responses. Individuals who had been on cART for less than 6 months were excluded from the study, as the minimum time required to determine response was 6 months.^8^

## Methodology

### Study population and design

A retrospective review was performed on the database (electronic and paper records) of Thohoyandou Community Health Centre (TCHC), which has one of the highest number of patients on cART in Vhembe District. TCHC is located in Thulamela Municipality in Vhembe District, which is the northernmost district in Limpopo Province, South Africa. It is a public health facility. A data collection form was designed to retrieve the data, including age, sex, marital status, year of initiation, baseline body mass index (BMI), HIV clinical stage, haemoglobin and estimated glomerular filtration rate (eGFR), along with the serial CD4+ cell counts and VL measurements. The study population included individuals initiated on cART between 01 January 2004 and 31 July 2016 and stored on tier.net. This included those transferred into the facility and those initiated at the facility. The database (tier.net) also had some missing data. Many patients did not have any VL and/or CD4 cell count results recorded even after 6 months, and as a result they were excluded from the study. Also excluded were those who defaulted and reinitiated later. Therefore, their duration on cART could not be correctly determined.

Typical first-line antiretroviral regimen consisted of stavudine, lamivudine and nevirapine or efavirenz, prior to 2010. Stavudine was replaced with tenofovir after 2010, with a fixed-dose combination (FDC) introduced in 2012. Second-line highly active antiretroviral therapy (HAART) consisted of abacavir or zidovudine, combined with lamivudine and ritonavir-boosted lopinavir. Third-line regimen was to be decided by a review committee.^8,17,18^

### Outcomes

#### Immunologic and virologic response

Virologic and immunologic responses were measured up to 132 months after cART initiation. Virologic response was measured using plasma HIV RNA concentrations. A VL of less than 50 copies/mL was considered suppressed. Because of differences in types of VL assays used over the period under review, analysis was also done using a VL less than 400 copies/mL as viral suppression. Immunologic response was measured by CD4+ cell count. An increase in CD4+ cell count of at least 50 cells/µL at 6 months after cART initiation was considered as adequate.^19^ If an individual had more than one VL or CD4+ cell count measurement at a fixed interval, the result closest to the particular interval was used. The sustainability of viral suppression was also reviewed. This was defined as having at least two consecutive VL results that were at most 50 copies/mL. The association of other covariates with these responses was also evaluated.

#### Mortality

Mortality was determined exclusively as recorded on tier.net. However, the recording of death on tier.net was not corroborated with national mortality database. This may have affected the validity of a more detailed analysis.

### Statistical analysis

The data used for the study were extracted from tier.net. Data not found on tier.net were retrieved from paper folders of individual patients. It was converted, using Microsoft Excel 2010, into a format for analysis in SPSS software version 24.0. Descriptive and inferential statistics were generated. During data cleaning, duplicate entries were identified and removed. The data set for socio-demographic variables and clinical variables were summarised. Continuous variables were presented as means. Categorical variables were presented as percentages or proportions. Mann–Whitney *U* test was used to check for equality of the median of the continuous variables (duration on cART and CD4+ cell count) as they were not normally distributed. Pearson’s chi-square test was used for categorical variables.

To model for factors associated with virologic and immunologic responses, univariate and multivariate logistic regression analysis was performed. Univariate analysis was performed first, and crude odds ratios for the association of the variables with immunologic and virologic responses determined. The variables included age, year of initiation, gender, marital status, baseline BMI, haemoglobin, clinical stage and estimated GFR. Variables with *p*-values < 0.2 were introduced into the multivariate models, and their adjusted odd ratios derived. The estimates used were 95% confidence interval (CI), and a *p*-value < 0.05 was considered to be statistically significant.

### Validity of instrument and reliability of the instrument

Face validity was ensured by conducting a review of a sample of the data collection form by non-experts in the field of HIV. Content validity was ensured by carrying out a detailed literature review and reviewing the instrument with an expert in the field of HIV research. Corrections were made based on the reviews.

The reliability of the data collection form was measured using interrater reliability method of reliability testing. The calculated Cohen κ coefficient was 0.79, which was within the acceptable limit.^20^

## Ethical consideration

Ethical clearance was obtained from the University of Venda Research Ethics Committee (Protocol number SHS/16/PH/16/1808). Approval was obtained from the Limpopo Provincial Department of Health to acquire and use the data for the study. The data stored on the computer were protected from theft using the most recent spyware protection.

## Results

### Socio-demographic and clinical characteristics

The clinical data of 1912 individuals were reviewed. Two hundred and seventy-nine individuals were excluded because they had been on treatment for less than 6 months. Of these, 68 had died. Those excluded for having no results even after 6 months were 177. Then, 209 individuals were not included because they defaulted treatment and were later reinitiated on cART. As a result, the determination of their duration on cART was affected. The clinical data of the remaining 1247 individuals were used for the analysis. Of these, 191 individuals initiated treatment in 2013. This corresponded to 15.4% of the total sample. One hundred and eighty-four individuals (14.8%) were initiated in 2014 and 183 (14.7%) in 2015. Only 0.3% of the cohort were initiated in 2004.

Twenty-four per cent of the cohort were male and the mean age was 36 ± 5 years. Other baseline characteristics of participants are shown in Table 1. Only 0.9% of the cohort had some form of post-exposure prophylaxis (PEP). Further review showed that 9.8% demised, 10.4% were lost to follow-up (LTFU), 11.1% transferred out and 68.7% were retained in care. Fourteen per cent of males demised during the period under review, as compared to 8.4% of females. This was found to be statistically significant (*p* = 0.01). There was no gender difference for those who were LTFU or who defaulted. Throughout the study, at least 98% of the cohort were on a first-line regimen. There was no one on a third-line regimen throughout the period under review.

**TABLE 1:** Socio-demographic and clinical characteristics of cohort.

Characteristics	Total	Percentage	*N*
**Gender**			
Male	307	24.6	1247
Female	940	75.4	
**Age (years)**			
< 15	67	5.4	1237
16–29	308	24.9	
30–44	582	47	
45–60	262	21.1	
> 60	19	1.5	
**Marital status**			
Married	326	45.3	719
Single	362	50.3	
Divorced	4	0.6	
Widowed	27	3.8	
**ART exposure**			
PMTCT	8	0.6	1236
PEP	2	0.2	
HAART	1	0.1	
None	1225	99.1	
**Baseline clinical stage**			
I	650	52.6	1236
II	176	14.2	
III	104	8.4	
IV	23	1.9	
Unknown	283	22.9	
**Baseline BMI**			
< 18.5	2	13.3	15
18.5–24.9	5	33.3	
25–29.9	4	26.7	
≥ 30	4	26.7	
**Baseline haemoglobin (g/dL)**			
< 6	4	1.1	356
6–9.9	59	16.6	
10–13	204	57.3	
> 13	89	25	
**Baseline eGFR (mL/min/1.73 m^2^)**			
16–29	1	0.2	436
30–59	41	9.4	
60–89	137	31.4	
≥ 90	257	58.9	
**Patient outcomes**			
Transfer out	137	11.1	1235
Still on treatment	848	68.7	
Demised	122	9.8	
Lost to follow-up	128	10.4	

ART, antiretroviral therapy; PMTCT, prevention of mother-to-child transmission; PEP, post-exposure prophylaxis; HAART, highly active antiretroviral therapy; BMI, body mass index; eGFR, estimated glomerular filtration rate.

### Virologic response

**FIGURE 1:**
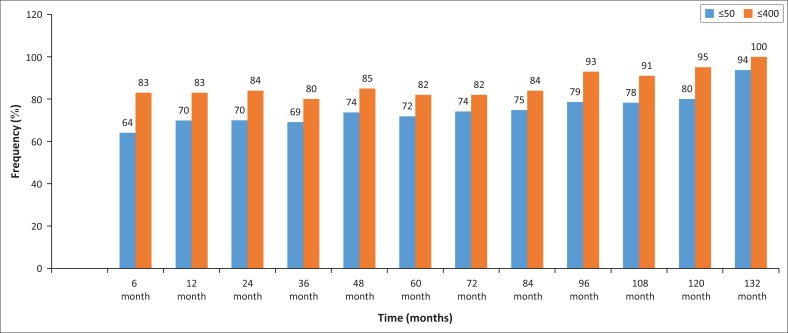
Proportion of patients with suppressed viral loads (using < 50 copies/mL and < 400 copies/mL) at fixed intervals.

After 6 months of cART, 64% (*n* = 648) were virally suppressed. At 12 months, 70% (*n* = 577) were suppressed, 70% (*n* = 453) at 24 months and 69% (*n* = 392) at 36 months. The proportion virally suppressed almost consistently increased with duration on cART. At 132 months, 94% (*n* = 16) were suppressed. When the definition of viral suppression was changed to ≤ 400 copies/mL, at least 80% viral suppression was attained at every interval throughout the study (Figure 1). Those with consecutive virally suppressed results were reviewed. The proportion with at least two consecutive suppressed VLs was 59% (*N* = 882). Only 14% had consistent viral suppression over a 54-month period. Twenty-eight (2.3%) had two or more VLs >1000 copies/mL at least 6 months apart and still remained on the failing regimen.

### Immunologic responses

**FIGURE 2:**
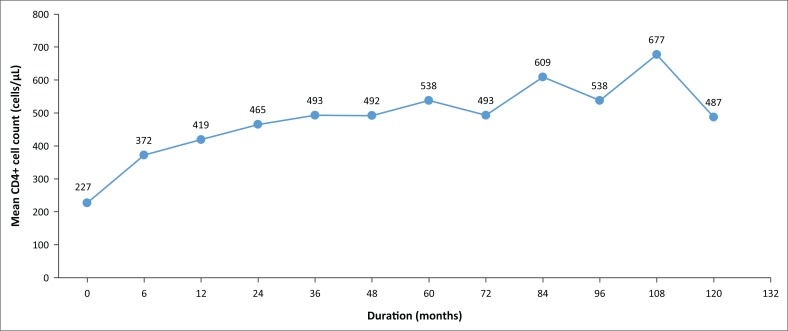
The mean CD4+ cell counts at fixed intervals over the time among the study population.

The mean CD4+ cell counts at specific times after initiation on cART from baseline were calculated. The mean CD4+ cell count at baseline was 227 cells/µL. This increased to 372 cells/µL at 6 months. The increases were consistent until at 60 months after initiation, at which point it was 538 cells/µL. At 120 months, the mean CD4+ cell count was 487 cells/µL (Figure 2). Fifty-five per cent had a CD4 cell count below 200 cells/µL at baseline. This proportion decreased to 26% after 6 months and 11% after 60 months. However, only 47% achieved a CD4+ cell count ≥ 500 cells/µL after 60 months on cART. Furthermore, there was no significant difference (*p* = 0.952) in achieving CD4+ cell count of ≥ 500 cells/µL at 60 months, when individuals with a baseline CD4+ cell count of ≤ 200 cells/µL were compared with those with a baseline CD4+ cell count > 200 cells/µL. The calculated immuno-virologic discordance for this cohort was 27% (*n* = 415). Also, of those that achieved viral suppression at 60 months, 56.4% achieved a CD4+ cell count.

### Factors associated with immunologic and virologic responses and mortality

Table 2 depicts the univariate and multivariate regression analysis for baseline factors associated with viral suppression. Univariate analysis showed that for every unit increase in haemoglobin, the odds of attaining viral suppression increase by 9%. Other factors significantly associated with viral suppression are age (31–60 years versus ≤ 15 years), gender (female) and WHO clinical stage II and III versus stage I. These findings were similar when multivariate logistic regression was performed. Multivariate analysis showed that the age group 31–45 years had 14% higher odds (adjusted odds ratio [aOR]: 1.14, 95% CI: 1.05–1.20; *p* = 0.046) of attaining at least one consecutive viral suppression, as compared to those less than 15 years. In addition, males had 38% lower odds (aOR: 0.62, 95% CI: 0.58–0.66; *p* = 0.011) of attaining viral suppression, as compared to females.

**TABLE 2:** Factors associated with having at least two consecutive suppressed viral loads.

Variable	Univariate			Multivariate		
	OR	95% CI	*p*	aOR	95% CI	*p*
**Age (years)**						
< 15	1	1	1	1	1	1
16–30	1.16	1.07–1.24	0.064	1.08	1.04–1.12	0.101
31–45	1.31	1.18–1.33	0.031	1.14	1.05–1.20	0.046
46–60	1.39	1.30–1.58	0.005	1.28	1.21–1.35	0.004
> 60	0.58	0.48–0.70	0.112	0.66	0.57–0.76	0.080
**Marital status**						
Unmarried	1	1	1	1	1	1
Married	0.53	0.48–0.59	0.131	0.73	0.69–0.79	0.083
Divorced	0.87	0.84–0.91	0.127	0.80	0.78–0.82	0.143
Widowed	1.82	1.74–1.90	0.026	1.78	1.71–1.85	0.112
**Gender (male)**	0.49	0.45–0.54	0.036	0.62	0.58–0.66	0.011
**Baseline clinical stage**						
I	1	1	1	1	1	1
II	0.92	0.89–0.96	0.019	0.61	0.58–0.67	<0.001
III	0.66	0.58–0.76	0.042	0.28	0.25–0.31	0.022
IV	0.54	0.50–0.60	0.063	0.26	0.18–0. 34	0.001
**Baseline haemoglobin (g/dL)**	1.09	1.01–1.16	0.043	1.39	1.30–1.48	0.048
**Previous ARV exposure (exposed)**	1.73	1.71–1.76	0.089	1.68	1.63–1.75	0.104
**Baseline CD4 count (per 10 cells/µL)**	1.34	1.29–1.40	0.143	1.12	1.08–1.16	0.108
**Duration on HAART (months)**	0.76	0.71–0.84	0.181	1.98	1.92–2.04	0.099

ARV, antiretroviral; HAART, highly active antiretroviral therapy; OR, odds ratio; aOR, adjusted odds ratio; CI, confidence interval.

The factors associated with adequate CD4+ cell count response, as shown in Table 3, were age and gender. Multivariate analysis showed that the age group 31–45 years had 10% higher odds (aOR: 1.10, 95% CI: 1.08–1.12; *p* = 0.01) of attaining adequate immunological response, as compared to those less than 15 years.

**TABLE 3:** Factors associated with adequate immunologic response.

Variable	Univariate			Multivariate		
	OR	95% CI	*p*	aOR	95% CI	*p*
**Age (years)**						
< 15	1	1	1	1	1	1
16–30	1.03	1.01–1.05	0.001	1.03	1.01–1.05	0.001
31–45	1.14	1.10–1.18	0.008	1.10	1.08–1.12	0.010
46–60	1.10	1.04–1.17	0.021	1.07	1.04–1.11	0.047
> 60	0.92	0.88–0.95	0.057	0.99	0.96–1.02	0.133
**Marital status**						
Unmarried	1	1	1	1	1	1
Married	1.34	1.14–1.57	0.110	1.44	1.26–1.64	0.133
Divorced	1.73	1.44–2.10	0.171	1.21	1.19–1.23	0.017
Widowed	2.02	1.79–2.28	0.121	2.47	2.27–2.68	0.101
**Gender (female)**	1.44	1.26–1.64	0.004	1.38	1.26–1.48	<0.001
**Baseline clinical stage**						
I	1	1	1	1	1	1
II	0.99	0.99–0.99	0.103	1.81	1.68–1.97	0.077
III	0.80	0.78–0.83	0.142	0.91	0.86–1.04	0.183
IV	0.88	0.80–0.97	0.101	1.08	0.98–1.13	0.073
Baseline haemoglobin (g/dL)	0.89	0.73–1.06	0.183	0.93	0.80–1.08	0.114
Previous ARV exposure (exposed)	3.47	3.22–3.54	0.198	2.88	2.80–2.93	0.085
Duration on HAART (months)	1.73	1.48–2.14	0.108	1.66	1.58–1.70	0.199

ARV, antiretroviral; HAART, highly active antiretroviral therapy; OR, odds ratio; aOR, adjusted odds ratio; CI, confidence interval.

## Discussion

This study reviewed virologic and immunologic responses of patients, in a rural community health centre in Vhembe District, South Africa, to cART over a 132-month period. It found that most (78.4%) patients were initiated on cART after 2010. This is probably because of changes in the South African NDoH guidelines since 2010, and the fact that patients were being initiated in any clinic closer to their homes. This increased uptake into the antiretroviral therapy programme.^21^

Furthermore, more females were initiated on HAART than males and most people in the cohort were aged 16–44 years. Johnson et al.^22^ found that women seemed to have more access to HAART than men and children. This is because pregnancy and breastfeeding are used as entry points for women by testing for HIV and initiation on cART. Presently, no such entry point exists for men and most children.

Our study also showed that only 0.9% of the cohort had any form of previous antiretroviral exposure. This proportion is quite low, considering that the guidelines recommending PEP have been available for more than 6 years. This may contribute to the high incidence of HIV in South Africa as, although PEP is not 100% effective, it has been shown to reduce HIV transmission in non-occupational exposures.^23^ However, data for previous exposure to PEP were obtained by recall of patients before being stored into the electronic database. Also, during transfer into the health facility, information on PEP was missing for some patients. This may account for the low prevalence of PEP exposure.

The results of this study showed a pattern in which the proportion of virally suppressed individuals increased with the passage of time. Also, the data recorded for individuals in this study also decreased consistently with time. This may have created a ‘retention bias’, which ensured that those who were likely to have unsuppressed VLs (defaulters, those that demised or were LTFU) did not have VL data used for these measurements. The proportion of individuals virologically suppressed was therefore calculated based on the number of individuals who had data recorded at a particular time point.

Our study found that 64% (*n* = 648), 70% (*n* = 577) and 72% (*n* = 366) of our cohort were virologically suppressed at 6, 12 and 60 months, respectively. When the definition of viral suppression was changed to < 400 copies/mL, the minimum proportion of virologically suppressed individuals at every time point throughout the study was 80%. This was similar to a large meta-analysis conducted by Boender and colleagues in low- to medium-income countries. They found that at every interval between 6 and 60 months, at least 80% of patients were virally suppressed.^24^ This was an unexpected but important finding in the drive towards achieving one of the ‘90-90-90 targets’ set by the UNAIDS. Present cART guidelines recommend initiation once diagnosed with HIV. One of the major aims of this is to prevent HIV transmission through achieving viral suppression, as revealed by the Partner study.^6^ A review was, therefore, attempted to assess how consistently patients were able to maintain viral suppression after attainment. Fifty-nine per cent of the sample were able to maintain one consecutive viral suppression. These patients maintained viral suppression over a period of at least 6 months. When reviewed for those able to maintain viral suppression consecutively for at least 54 months, only 14% of the sample achieved this. This low proportion of people with consistent viral suppression may have a negative impact on the use of viral suppression as a prevention strategy.

With the initiation of cART, immune recovery, in the form of CD4+ cell count increase, is expected. In our review of immunological response to cART, the mean CD4+ cell count increased almost throughout the study, as was the case in a study in Tshwane District. However, their cohort did not achieve a normal CD4+ cell count (> 500 cells/µL) at 60 months, unlike in our study.^25^ Also, at 60 months, only 11% of patients had a CD4+ cell count of less than 200 cells/µL. This meant that fewer patients may have needed prophylaxis against opportunistic infections. It must, however, be stated that the baseline CD4+ cell count of many patients initiated prior to 2010 was missing on tier.net.

The only factors found to be significantly associated with early viral suppression were baseline CD4 count and gender, with individuals with higher CD4+ cell counts and females more likely to achieve viral suppression at 6 months. The age group less than 15 years also had comparatively poorer immunological and virologic outcomes. Problems with adherence and drug formulations may account for this. Mlangeni and Senkubuge,^25^ in a study to ascertain patient retention on ART after 5 years of treatment in Tshwane District, however, found that gender and age had no influence on early VL response (6 months). Some other studies also found that higher baseline CD4+ cell count was associated with viral suppression, and males, age group less than 15 years and prior ART exposure were more likely to experience virologic failure.^13,14^ When the factors that are associated with viral suppression in the long term were reviewed, gender and baseline laboratory results had no significant association. It seemed as if males that were able to stay on treatment for longer had similar virologic response to females. This may have been because of the retention bias, noted above, as more males died or were LTFU during the study. Bello et al.^16^ also found that absence of prior exposure to antiretrovirals before initiation on lifelong HAART was associated with viral suppression at 10 years post-initiation. They also found that age, race and baseline CD4+ cell count had no influence on viral suppression in the longer term.

### Limitations of the study

Although attempts were made to prevent bias, missing data on tier.net and the use of patient recall to record some variables, such as previous antiretroviral therapy exposure, may have introduced some bias to the study. Furthermore, patients recorded as LTFU may have died. However, because the data were not updated with the South African national mortality database, the cumulative mortality may have been underestimated. Furthermore, being a retrospective study, data on some variables that may influence responses to cART, such as adherence, were not available. This introduces the possibility of residual confounding into the study.

Despite the limitations outlined above, this study has a number of strengths. The use of all patients on cART recorded on tier.net affords an opportunity to find a difference in outcomes if there really is one. Finally, there is a paucity of studies reviewing immunologic and virologic responses in the long term in healthcare facilities in Limpopo, South Africa; therefore, this study will serve as a foundational study for such research.

## Conclusion

According to the South African NDoH definition for viral suppression, the proportion of individuals attaining viral suppression was well below the UNAIDS 90% target. Also, the sustainability of viral suppression seemed quite low when individuals were reviewed over a 54-month period. Immunologic response was adequate for most individuals in the period under review. Being male was associated with poorer virologic response to cART. Furthermore, the age group less than 15 years had poorer immunologic and virologic outcomes. Therefore, a targeted approach focusing on improving virologic and immunologic responses of males and patients less than 15 years may aid in improving outcomes at a community level because infected male partners are likely to infect a female partner and vice versa.^26^
